# Gaps in awareness and clinical practices regarding GERD among healthcare physicians in Pakistan

**DOI:** 10.12669/pjms.42.5.13387

**Published:** 2026-05

**Authors:** Ghias Un Nabi Tayyab, Saad Khalid Niaz, Zaigham Abbas, Sher Rehman, Rafi Ud Din, Daud Ghilzai, Mahaveer Maheshwari

**Affiliations:** 1 Prof. Dr. Ghias Un Nabi Tayyab, MBBS, FCPS, MRCP, FRCP, AGAF,Dean, Faculty of Gastroenterology,College of Physicians and Surgeons of Pakistan, Karachi, Pakistan; 2 Prof. Dr. Saad Khalid Niaz, MBBS, MRCP, FRCP.Sindh Institute of Advanced Endoscopy and Gastroenterology (SIAG), Karachi, Pakistan; 3 Prof. Dr. Zaigham Abbas, MBBS, FCPS, FRCPDr. Ziauddin University Hospital, Clifton Karachi, Pakistan; 4 Prof. Dr. Sher Rehman, MBBS, FCPSKhyber Girls’ Medical College, Peshawar, Pakistan; 5 Prof. Dr. Rafi Ud Din, MBBS, FCPS, FRCPCombined Military Hospital Lahore,Medical College and Institute of Dentistry, Lahore, Pakistan; 6 Dr. Daud Ghilzai, MBBS, FCPSBolan Medical College, Quetta, Pakistan; 7 Dr. Mahaveer MaheshwariLiaquat University of Medical and Health Sciences, Jamshoro, Pakistan

**Keywords:** Attitudes, Gastroesophageal Reflux, Knowledge, Pakistan, Practice, Physicians

## Abstract

**Objective:**

To assess the knowledge, understanding, clinical practices, and challenges of physicians in Pakistan concerning Gastroesophageal reflux disease (GERD), and to identify areas for improvement to enhance patient outcomes.

**Methodology:**

This cross-sectional survey was conducted among 435 physicians across Pakistan, including general practitioners, gastroenterologists, and internal medicine specialists. A structured, self-administered questionnaire collected data on demographics, knowledge of GERD risk factors and symptoms, diagnostic practices, management approaches, and perceived challenges.

**Results:**

Among 435 surveyed physicians, 80.2% reported being very familiar with GERD diagnosis and management, yet there were notable gaps in recognizing atypical and extra-esophageal symptoms. While acid regurgitation (91.5%) and heartburn (82.8%) were the most commonly recognized symptoms, less frequent acknowledgment was found for dysphagia (42.8%), cough (47.6%), and sore throat (37.7%). Only 14.0% of respondents consistently considered extra-esophageal symptoms in the context of GERD. Additionally, risk factors such as diet (82.3%), obesity (78.9%), and smoking (77.5%) were widely recognized, but challenges like poor treatment adherence (73.1%) and medication costs (46.0%) were reported. A majority of respondents requested more training (61.8%) and availability of updated national guidelines (57.2%). These findings highlight gaps in symptom recognition and the need for enhanced education and research to improve GERD diagnosis and management practices.

**Conclusion:**

While most physicians demonstrated adequate knowledge of GERD, significant gaps in recognizing atypical symptoms, utilization of advanced diagnostics, and addressing management challenges were noted. Tailored interventions, including updated national GERD guidelines, focused training programs, and enhanced access to diagnostic and management tools, are essential to improving care quality and patient outcomes in Pakistan.

## INTRODUCTION

Gastroesophageal reflux disease (GERD) is a prevalent condition globally, marked by symptoms such as heartburn and regurgitation, caused by the reflux of gastric contents into the esophagus. Its incidence has increased and is now considered to be one of the most frequent diagnoses both in primary care physicians and gastroenterology specialists.^1^ GERD is frequently difficult to diagnose since symptoms are often non-specific and overlap with other gastrointestinal, cardiac, and respiratory conditions such as functional dyspepsia, ischemic heart disease, chronic cough, asthma and laryngopharyngeal disorders.^2^ Consequently, patients can show atypical or extra-esophagic manifestations, which results in delayed or incorrect diagnosis. Moreover, treatment of GERD is complicated especially in the advanced stages or when the condition has complications, including Barrett esophagus and esophageal adenocarcinoma that demand prolonged monitoring and specific treatment interventions.^3^,^4^

The prevalence of GERD is increasing due to multiple contributing factors, including rising obesity rates, an aging population, and significant shifts in lifestyle pattens.^5^ In primary care, the diagnosis of GERD is primarily based on the clinical presentation of symptoms such as heartburn, regurgitation, and chest pain; however, this symptom-based approach has inherent limitations. Esophagogastroduodenoscopy (EGD) is often preferred in complicated cases, while 24-hour pH-metry is utilized occasionally.^6^

Lifestyle changes, patient education and pharmacotherapy are foundations for the management of GERD. Proton pump inhibitors (PPIs) are still used as first line therapy, but they have side effects, and do not cure the condition in some patients on long term therapy.^7^-^11^ The most common side effects included an increased risk of bone fractures (23.9%) and cardiovascular risk (21.7%).^12^ Shared decision-making and patient-centered communication are essential to increase treatment compliance and satisfaction.^13^,^14^

Studies reveal significant gaps in knowledge and varying practices among healthcare providers. Factors such as specialty training, access to guidelines, and clinical experience impact their ability to manage GERD effectively.^4^,^15^,^16^ Although locally developed clinical practice guidelines for gastroesophageal reflux disease (GERD) are available,^17^ a substantial gap persists between both national and international guidelines and their consistent application in routine clinical practice.

This study aimed to explore the knowledge, understanding, and specific challenges of physicians in Pakistan regarding GERD. By identifying gaps and challenges, the findings aim to build strategies to improve clinical practices and optimize patient care outcomes.

## METHODOLOGY

This study utilized a cross-sectional survey design to assess the knowledge, understanding, and needs of physicians concerning gastroesophageal reflux disease (GERD) in Pakistan. The survey was conducted among general practitioners, gastroenterologists and internal medicine specialists to provide a broad spectrum of perceptions of GERD diagnosis, treatment and management. The data was collected from different health related facilities in Pakistan including government health facilities and private clinics and community health centers from August to November 2024.

### Ethical consideration:

The ethical approval was obtained from the Institutional Review Committee of Bahira International Hospital Orchard, Lahore Pakistan [Ref # IRB/BIHO/2024/07-C ; Dated 15^th^ May 2024].

### Participants:

A total of 435 physicians were enrolled, including both male and female participants. Eligible participants were licensed practitioners in Pakistan who were actively engaged in diagnosing and managing GERD patients while those who were not involved in GERD management were excluded from the study.

### Sampling method:

A convenience sampling method was applied. Physicians who were available and willing to participate were selected from various healthcare facilities.

### Informed consent:

The study’s purpose was explained and written informed consent was obtained from all participants, prior enrollment. Involvement was voluntary, and the subjects were free to pull out at any time.

### Variables and data collection:

A structured questionnaire was used for data collection, which was distributed both electronically and in paper format. The questionnaire was administered in English. The questionnaire was adapted from previously published physician-based surveys assessing knowledge, attitudes, and clinical practices related to gastroesophageal reflux disease (GERD) and was modified to reflect the local healthcare context in Pakistan. Its development was guided by international GERD management guidelines and established principles for healthcare questionnaire design. The Knowledge-related questions focused on physicians’ familiarity with GERD diagnosis, risk factors, diagnostic tests, and treatment options. Clinical practice-related questions assessed the frequency of GERD consultations, management strategies for refractory cases, and treatment approaches. The questionnaire also explored the physicians’ perceptions of the need for updated GERD guidelines, additional resources, and training opportunities. To ensure content validity, the adapted questionnaire underwent expert review by senior gastroenterologists, and a pilot assessment was conducted including 30 physicians to evaluate comprehensibility and relevance. The questions were adjusted as per the input received from the panel.

### Statistical Analysis:

SPSS version 25.0 was used for statistical analysis. Descriptive statistics was used to summarize the demographic characteristics of the participants and the distribution of responses to the knowledge, clinical practice, and needs of physician regarding GERD.

## RESULTS

The demographic and professional characteristics of the survey respondents are summarized in Table-I. The age distribution of the respondents shows that the majority (57.5%) were between 25 and 35 years old, followed by 26.4% aged between 36 and 45 years. Fewer respondents were in the 46-55 age group (10.3%), and 5.7% were aged 56 or older. The gender distribution revealed a predominance of male respondents, who constituted 83.9%, while females represented 16.1% of the sample. In terms of geographical location, 75.2% of the physicians practiced in urban areas, whereas 24.8% worked in rural settings. The primary practice setting varied, with 55.2% working in government hospitals, 39.8% in private clinics/hospitals, 0.7% in community health centers, and 4.4% in other settings. Regarding specialization, the majority of respondents were general practitioners (40.9%), followed by internal medicine specialists (23.7%), gastroenterologists (18.6%), and other specialists (16.8%).

**Table-I:** Demographic and Professional Characteristics of Survey Respondents.

Variables		n (%)
Age	25-35	250 (57.5%)
	36-45	115 (26.4%)
	46-55	45 (10.3%)
	56 and above	25 (5.7%)
Gender	Female	70 (16.1%)
	Male	365 (83.9%)
Location	Rural	108 (24.8%)
	Urban	327 (75.2%)
Primary Practice Setting	Community Health Center	3 (0.7%)
	Government Hospital	240 (55.2%)
	Other	19 (4.4%)
	Private Clinic/Hospital	173 (39.8%)
Specialization	Gastroenterologist	81 (18.6%)
	General Practitioner	178 (40.9%)
	Internal Medicine	103 (23.7%)
	Other	73 (16.8%)

The physicians’ knowledge and understanding of GERD is shown in Table-II. A vast majority of respondents reported being very familiar with the diagnosis and management of GERD. But lacked in the basic recognition of symptoms, risk factors etc. Diet was identified as the pre-dominant risk factor (82.3%) followed by obesity (78.9%) and smoking (77.5%). The most commonly associated symptoms of GERD included acid regurgitation (91.5%), followed by heartburn (82.8%). In terms of diagnostic tests, clinical history alone was used by 59.3% of respondents, followed by upper endoscopy (49.0%) and 24-hour pH monitoring (44.8%). Extraesophageal symptoms were considered related to GERD “sometimes” by 69.7% of physicians. Lifestyle modifications, including diet and weight loss, were strongly agreed upon as important by 66.4% of respondents. Regarding treatment effectiveness, 60.0% found Proton Pump Inhibitors (PPIs) very effective, while 41.4% regarded Potassium channel blockers (PCABs) as very effective. A majority (68.5%) were very familiar with other GERD medications, such as H2 blockers and prokinetics.

**Table-II:** Knowledge and understanding of GERD.

Questions		N (%)
How familiar are you with the diagnosis and management of GERD?	Not familiar	14 (3.2%)
	Somewhat familiar	72 (16.6%)
	Very familiar	349 (80.2%)
*Which of the following do you consider risk factors for GERD?	Alcohol Consumption	299 (68.7%)
	Smoking	337 (77.5%)
	Diet	358 (82.3%)
	Obesity	343 (78.9%)
	Pregnancy	230 (52.9%)
	Other	95 (21.8%)
*What symptoms do you primarily associate with GERD?	Heartburn	360 (82.8%)
	Acid Regurgitation	398 (91.5%)
	Chest Pain	243 (55.9%)
	Dysphagia (difficulty swallowing)	186 (42.8%)
	Cough	207 (47.6%)
	Sore Throat	164 (37.7%)
*Which diagnostic tests do you typically use for GERD patients?	Clinical history alone	258 (59.3%)
	Upper endoscopy (EGD)	213 (49.0%)
	24-hour pH monitoring	195 (44.8%)
	Esophageal manometry	87 (20.0%)
	Bravo capsule monitoring	21 (4.8%)
	Other	31 (7.1%)
How often do you consider extraesophageal symptoms (e.g., cough, asthma, laryngitis) as related to GERD?	Always	61 (14.0%)
	Never	14 (3.2%)
	Rarely	57 (13.1%)
	Sometimes	303 (69.7%)
Do you believe lifestyle modifications (e.g., diet, weight loss) are important in managing GERD?	Agree	111 (25.5%)
	Disagree	1 (0.2%)
	Neutral	7 (1.6%)
	Strongly agree	289 (66.4%)
	Strongly disagree	27 (6.2%)
In your opinion, how effective are Proton Pump Inhibitors (PPIs) in managing GERD?	Ineffective	9 (2.1%)
	Moderately effective	148 (34.0%)
	Slightly effective	17 (3.9%)
	Very effective	261 (60.0%)
In your opinion, how effective are Potassium channel blockers (PCABs) in managing GERD?	Ineffective	25 (5.7%)
	Moderately effective	157 (36.0%)
	Slightly effective	73 (16.8%)
	Very effective	180 (41.4%)
How familiar are you with other GERD medications besides PPIs? (e.g., H2 blockers, antacids, prokinetics, PCABs)	Not familiar	18 (4.1%)
	Somewhat familiar	119 (27.4%)
	Very familiar	298 (68.5%)

* Participants were allowed to select more than one response. Percentages are calculated based on the total number of respondents (N = 435) and may exceed 100% due to multiple selections.

The clinical practices and challenges faced by physicians in managing GERD are highlighted in Table-III. A significant portion (63.9%) of respondents provided daily consultations for GERD patients, with 22.5% consulting weekly, 8.7% monthly, and 4.8% rarely. Pediatric GERD cases were encountered by 41.8% of respondents, while 58.2% reported not managing these cases. The challenges most commonly faced in managing GERD included poor patient adherence to treatment (73.1%), patient misconceptions (49.2%) and high cost of medication (46.0%). In cases of non-response to initial treatments, 19.8% of physicians referred patients for endoscopy, 22.5% switched to PCABs, and 14.0% referred patients to specialists. A substantial majority (59.3%) screened for Helicobacter pylori in select cases, while 25.3% always did so, and 15.4% did not routinely screen.

**Table-III:** Clinical Practice and Challenges

Questions		N (%)
How often do you give consultation to GERD patients in your practice?	Daily	278 (63.9%)
	Monthly	38 (8.7%)
	Rarely	21 (4.8%)
	Weekly	98 (22.5%)
Do you encounter pediatric GERD cases in your practice?	No	253 (58.2%)
	Yes	182 (41.8%)
*What are the common challenges you face in managing GERD patients?	Accessibility of medications	82 (18.9%)
	Difficulty in diagnosing atypical cases	112 (25.7%)
	Poor patient adherence to treatment	318 (73.1%)
	High cost of medication	200 (46.0%)
	Limited diagnostic tools	173 (39.8%)
	Limited treatment options	68 (15.6%)
	Patient misconceptions	214 (49.2%)
	Other	36 (8.3%)
What is your main approach to patients who do not respond to initial GERD treatments (e.g., PPIs)?	Increase frequency OD to BID	63 (14.5%)
	Increase PPI dosage	1 (0.2%)
	Increase PPI dosage to high strength	60 (13.8%)
	Other	21 (4.8%)
	Refer for endoscopy	86 (19.8%)
	Refer to a specialist	61 (14.0%)
	Switch from PPI to other PPI	45 (10.3%)
	Switch from PPI to PCAB	98 (22.5%)
Do you screen for Helicobacter pylori in GERD patients?	No, not routinely	67 (15.4%)
	Yes, always	110 (25.3%)
	Yes, in select cases	258 (59.3%)

* Participants were allowed to select more than one response. Percentages are calculated based on the total number of respondents (N = 435) and may exceed 100% due to multiple selections.

The physicians’ needs and recommendations for improving GERD management in Pakistan are shown in Table-IV. A majority (61.8%) expressed the need for training or workshops on GERD management, followed by patient education materials (59.1%) and the desire for access to advanced diagnostic tools (55.6%). Additionally, 57.2% of respondents highlighted the need for updated national GERD guidelines. In terms of priorities for new national GERD guidelines, 72.0% of physicians emphasized focusing on lifestyle modifications, and 60.0% believed cost-effective treatments must be prioritized in new national guidelines. A significant 89.7% of respondents agreed on the necessity for more local research on GERD in Pakistan. Regarding telemedicine, 68% of respondents believed it would be helpful for managing GERD patients in remote areas, while 23% were unsure and 9% disagreed. A substantial 78.4% of respondents were interested in participating in future training sessions or workshops on GERD management.

**Table-IV:** Needs and Recommendations for GERD Management Guidelines

Questions		N (%)
*What resources or support would you like to improve your management of GERD patients?	Access to advanced diagnostic tools (e.g., pH monitoring)	242(55.6%)
	Patient education materials	257 (59.1%)
	Updated national GERD guidelines	249 (57.2%)
	Training or workshops on GERD management	269 (61.8%)
	Other	28(6.4%)
*What do you believe should be prioritized in new national GERD guidelines for Pakistan?	Cost-effective treatments	261 (60.0%)
	Focus on lifestyle modifications	313 (72.0%)
	Management of extraesophageal symptoms	170 (39.1%)
	Management of refractory GERD cases	244 (56.1%)
	Simplified diagnostic criteria	189 (43.4%)
	Use of alternative therapies (e.g., herbal medicine)	93 (21.4%)
	Other	30 (6.9%)
Would you be interested in participating in future training sessions or workshops on GERD management?	Maybe	82 (18.9%)
	No	12 (2.8%)
	Yes	341 (78.4%)
Do you feel the need for more local research on GERD in Pakistan?	Maybe	27 (6.2%)
	No	18 (4.1%)
	Yes	390 (89.7%)
Would you find telemedicine helpful for managing GERD patients in remote areas?	Maybe	100 (23%)
	No	39 (9%)
	Yes	296 (68%)

* Participants were allowed to select more than one response. Percentages are calculated based on the total number of respondents (N = 435) and may exceed 100% due to multiple selections.

## DISCUSSION

This study provides valuable insights into the knowledge, practices, challenges, and needs of physicians regarding GERD management in Pakistan. While a foundational understanding of the disease is evident among most respondents, several significant gaps were identified that highlight the urgent need for structured, updated national GERD guidelines. When compared with the existing Guidelines (2015)^17^, which emphasize comprehensive symptom recognition (including extra-esophageal manifestations), risk factor modification, structured diagnostic pathways, and evidence-based pharmacological management, the findings suggest only partial alignment between current clinical practice and national recommendations. Even though the majority of physicians expressed the awareness of the typical symptoms of GERD and supported the use of proton pump inhibitors as the first-line therapy in accordance with PSG-advocated guidelines, the lack of the understanding of atypical manifestations, the risk factors that can be altered, and the shift of treatment opportunities signifies the gaps in the awareness of the guidelines, their dissemination, and incorporation. These inconsistencies emphasize the necessity to revise national GERD guidelines to accurately represent the current evidence and promote their implementation in regular clinical practice with the help of specific training and continuing medical education programs.

Although 80.2% of respondents reported being ‘very familiar’ with GERD diagnosis and management, but there is under-recognition of extra-esophageal and atypical symptoms. For instance, although acid regurgitation (91.5%) and heartburn (82.8%) were correctly identified as cardinal symptoms, other relevant symptoms such as dysphagia (42.8%), cough (47.6%), and sore throat (37.7%) were less frequently recognized. Similarly, other studies also suggest that physicians’ knowledge of GERD management is limited, with only 26% of residents and 37% of consultants demonstrating good knowledge scores.^4^ Comparatively, Alzahrani and Al Turki (2021) reported a moderate knowledge level among family and internal medicine residents in Riyadh, with a median knowledge score of 62.5%.^17^ Moreover, extraesophageal symptoms were inconsistently acknowledged, with only 14.0% considering them always in relation to GERD, which is in contrasts with the emphasis in international literature on these symptoms as significant diagnostic indicators.^2^,^18^,^19^

In terms of risk factors, although dietary habits (82.3%), obesity (78.9%), and smoking (77.5%) were widely acknowledged, fewer respondents identified alcohol consumption (68.7%) and pregnancy (52.9%) as significant contributors. These findings are also consistent with the exiting literature,^20^-^23^ modifiable factors such as obesity, smoking, alcohol consumption, and certain dietary habits are among the most common contributors to GERD development and progression.

Diagnostic practices in Pakistan show a preference for clinical history and endoscopy. Moreover, while 60.0% of physicians regarded PPIs as very effective, there was notable uncertainty regarding the efficacy of newer therapies such as PCABs. This preference mirrors findings by Klenzak et al. (2018),^2^ where PPIs were described as the preferred first-line treatment despite concerns over long-term side effects and potential overuse.^7^-^11^ Despite evidence in the existing literature showing that PCABs are comparable to PPIs in treating GERD and may offer superior symptom relief especially in PPI-refractory cases physicians’ limited confidence in these alternatives suggests a knowledge gap.^24^

Challenges in clinical practice further highlight knowledge and implementation gaps. Poor patient adherence (73.1%), patient misconceptions (49.2%), and high medication costs (46.0%) were major barriers identified by physicians. Similar findings are reported in other studies.^4^,^25^ However, barriers to effective GERD management in some regions include lack of local guidelines, limited region-specific research, and adherence to traditional practices.^26^ Patient-physician communication is crucial in GERD management, as treatment goals focus on symptom reduction.^27^

A significant proportion of respondents expressed the need for training workshops (61.8%), access to advanced diagnostics (55.6%), and patient education materials (59.1%). Furthermore, 57.2% of physicians called for updated national GERD guidelines, with specific emphasis on lifestyle modification (72.0%), cost-effective treatments (60.0%), and simplified diagnostic criteria (43.4%). The desire for such resources underscores the perceived gaps in current national standards and the fragmented approach to GERD care. Importantly, nearly 90% of respondents agreed on the need for more local research on GERD, indicating limited existing data to guide context-specific decision-making in Pakistan.

Overall, this study underscores a significant gap in physician awareness regarding GERD symptomatology, risk factors, and comprehensive management strategies. Despite self-reported familiarity, the inconsistencies in recognizing cardinal and extraesophageal symptoms, as well as modifiable risk factors like obesity, highlight a disconnect between perceived and actual knowledge. These findings, alongside international comparisons, strongly support the urgent need for structured, evidence-based national GERD guidelines in Pakistan. Such guidelines, complemented by continuous medical education (CME) and training programs, could improve diagnostic accuracy, promote appropriate treatment choices, and encourage the integration of biopsychosocial models into routine clinical care. Addressing these gaps is essential not only for enhancing physician competence but also for improving patient outcomes in GERD management.

### Limitations:

This study provides valuable insights into GERD management among physicians in Pakistan, yet several limitations must be acknowledged. First, the study sample demonstrated a marked gender imbalance, with a predominance of male respondents, which introduces potential gender-related bias. The differences in clinical training programmes, interactions with patients, and practice patterns between male and female physicians could have an impact on the diagnostic and management methods; thus, the results might not adequately represent the gender-diverse views on the management of GERD. Additionally, reliance on self-reported data may have introduced response bias, while the predominance of urban participants (75.2%) limits the generalizability to rural settings. Though patients suffering from GERD are managed in the primary care settings and mostly by senior family practitioners, most of the respondents are hospital doctors and are of younger age group indicating a possible lesser experience and expertise with GERD diagnosis and management. The cross-sectional design restricts causal inferences, and the exclusion of other healthcare professionals overlooks their roles in GERD management. Limited access to advanced diagnostics and therapies in some regions may have influenced reported practices, making findings less generalizable to resource-rich settings. Additionally, the study’s focus on physicians without patient outcome data limits its ability to assess the real-world effectiveness of management strategies. Lastly, voluntary participation could have led to sampling bias, potentially skewing results toward more engaged or knowledgeable respondents.

## CONCLUSION

This study highlights the knowledge, understanding, and clinical practices of physicians in Pakistan regarding GERD management, revealing both strengths and areas for improvement. While most physicians demonstrated familiarity with GERD diagnosis and treatment, gaps were identified in recognizing atypical symptoms, risk factors such as heartburn, obesity and pregnancy, and in the utilization of advanced diagnostic tools. Challenges, including patient adherence, high medication costs, and limited access to resources, were significant barriers to optimal care. The findings underscore the need for tailored interventions, such as updated national GERD guidelines, focused training programs, and enhanced access to diagnostic tools, particularly in rural settings. Increased awareness, coupled with localized research and patient-centered strategies, can improve GERD management and ultimately enhance patient outcomes in Pakistan.

### Author contributions:

**GUNT, SKN & MM** substantial contributions to conception and design of the study, acquisition of data, or analysis and interpretation of data;

**ZA, SR & DG** drafting the article or making critical revisions related to important intellectual content of the manuscript;

**GUNT & RUD** final approval of the version of the manuscript to be published.

**GUNT, SKN, ZA,SR, RUD, DG, MM -** Agreement to be accountable for all aspects of the work in ensuring that questions related to the accuracy or integrity of any part of the work are appropriately investigated and resolved.
